# Association between maternal stature and household-level double burden of malnutrition: findings from a comprehensive analysis of Ethiopian Demographic and Health Survey

**DOI:** 10.1186/s41043-023-00347-9

**Published:** 2023-01-24

**Authors:** Biniyam Sahiledengle, Lillian Mwanri, Kingsley Emwinyore Agho

**Affiliations:** 1 Department of Public Health, Madda Walabu University Goba Referral Hospital, Bale-Goba, Ethiopia; 2 Centre for Public Health Research, Equity and Human Flourishing, Torrens University Australia, Adelaide Campus, Adelaide, SA 5000 Australia; 3 School of Health Sciences, Western Sydney University, Locked Bag 1797, Penrith, NSW 2751 Australia; 4 School of Medicine, Translational Health Research Institute, Western Sydney University, Campbelltown Campus, Penrith, NSW 2571 Australia; 5 African Vision Research Institute, University of KwaZulu-Natal, Durban, 4041 South Africa

**Keywords:** Double burden of malnutrition, Dual forms of malnutrition, Ethiopia, Maternal stature, Mother–child pairs, Overweight mothers, Underweight child

## Abstract

**Background:**

Undernutrition among under-five children is one of the intractable public health problems in Ethiopia. More recently, Ethiopia faced a rising problem of the double burden of malnutrition—where a mother may be overweight/obese, and a child is stated as having undernutrition (i.e., stunting, wasting, or underweight) under the same roof. The burden of double burden of malnutrition (DBM) and its association with maternal height are not yet fully understood in low-income countries including Ethiopia. The current analysis sought: (a) to determine the prevalence of double burden of malnutrition (i.e., overweight/obese mother paired with her child having one form of undernutrition) and (b) to examine the associations between the double burden of malnutrition and maternal height among mother–child pairs in Ethiopia.

**Methods:**

We used population-representative cross-sectional pooled data from four rounds of the Ethiopia Demographic and Health Survey (EDHS), conducted between 2000 and 2016. In our analysis, we included children aged 0–59 months born to mothers aged 15–49 years. A total of 33,454 mother–child pairs from four waves of EDHS were included in this study. The burden of DBM was the primary outcome, while the maternal stature was the exposure of interest. Anthropometric data were collected from children and their mothers. Height-for-age (HFA), weight-for-height (WFH), and weight-for-age (WFA) *z*-scores < − 2 SD were calculated and classified as stunted, wasting, and underweight, respectively. The association between the double burden of malnutrition and maternal stature was examined using hierarchical multilevel modeling.

**Results:**

Overall, the prevalence of the double burden of malnutrition was 1.52% (95% CI 1.39–1.65). The prevalence of overweight/obese mothers and stunted children was 1.31% (95% CI 1.19–1.44), for overweight/obese mothers and wasted children, it was 0.23% (95% CI 0.18–0.28), and for overweight/obese mothers and underweight children, it was 0.58% (95% CI 0.51–0.66). Children whose mothers had tall stature (height ≥ 155.0 cm) were more likely to be in the double burden of malnutrition dyads than children whose mothers’ height ranged from 145 to 155 cm (AOR: 1.37, 95% CI 1.04–1.80). Similarly, the odds of the double burden of malnutrition was 2.98 times higher for children whose mothers had short stature (height < 145.0 cm) (AOR: 2.98, 95% CI 1.52–5.86) compared to those whose mothers had tall stature.

**Conclusions:**

The overall prevalence of double burden of malnutrition among mother–child pairs in Ethiopia was less than 2%. Mothers with short stature were more likely to suffer from the double burden of malnutrition. As a result, nutrition interventions targeting households’ level double burden of malnutrition should focus on mothers with short stature to address the nutritional problem of mother and their children, which also has long-term and intergenerational benefits.

## Introduction

The coexistence of overweight and undernutrition among the members of a single household known as the double burden of malnutrition (DBM) has drawn more attention in recent years [1]. Undernutrition and overnutrition coexist simultaneously despite previously being understood and treated as separate public health problems [2, 3]. According to the World Health Organization (WHO), the DBM is “characterized by the coexistence of undernutrition along with overnutrition (overweight and obesity), or diet-related non-communicable diseases, within individuals, households and populations, and across the life-course” [4]. At the household level, a double burden of malnutrition can exist—when a mother may be overweight/obese and a child has an undernutrition status (i.e., stunting, wasting, or underweight) [5].

Globally, around 45% of deaths among children under 5 years are linked to undernutrition [6]. It is also estimated that 149.2 million children under 5 suffered from stunting, while wasting affected 49 million children under the age of 5 in 2020 [6]. Evidence also showed that two out of five and more than one-quarter of all children suffering from stunting and wasting lived in Africa [6)]. Meanwhile, in sub-Saharan Africa (SSA), the prevalence of overweight and obesity is rising rapidly, with adult women bearing the greatest burdens—ranging from 5.6 to 27.7% [7]. A recent study examining the trends of overweight and obesity among women in Africa showed statistically significant increasing trends in several SSA countries [8]. Furthermore, due to the rapid ongoing global nutrition transition, an increasing number of studies demonstrate that the double burden of malnutrition (DBM) is a particular challenge for low- and middle-income countries (LMICs) [1, 9–17].

Although research is limited, sub-Saharan Africa has also been experiencing high levels of DBM in recent years [7, 18–20]. Earlier estimates on DBM in sub-Saharan Africa reported below 10% prevalence at the household level [21]. However, more recent studies have reported a high prevalence of DBM among mother–child pairs in SSA: overweight/obese mother–stunted child pairs, 13–20% in Kenya [22, 23], 10.3% in Nigeria [24], 14% in Egypt [21], and 1.8% to 23% in Ethiopia [11, 25, 26].

In Ethiopia, malnutrition affects women and children disproportionately [27, 28]. Overweight/obesity is rising rapidly while child undernutrition remains persistent. The prevalence of stunting, wasting, and being underweight were 37%, 21%, and 7%, respectively according to the 2019 Ethiopian Mini Demographic and Health Survey report [29]. Compared to the WHO cutoff values for the significance of undernutrition, the prevalence of stunting, wasting, and being underweight remained a serious public problem in the country [30].

Undernutrition has long been considered a major issue in Ethiopia; overweight and obesity have also been identified as growing problems [26, 31]. According to a recent study, 14.9% of women aged 15–49 years are overweight or obese, of which 83.3% were urban dwellers [32]. A recent systematic review and meta-analysis also reported that the estimated pooled prevalence of overweight and obesity among adults in Ethiopia was 20.4% and 5.4%, respectively [33]. It has been noted that mothers’ overweight/obesity is associated with the nutrition transition situation [34], due to a shift in dietary patterns with populations in developing countries consuming more energy dense than before due to changes in economic conditions. This demonstrated that Ethiopia, like other LMICs, is subject to the inevitable consequences of DBM; however, the burden of DBM is still not fully understood [25, 26]. So far, a few studies on overweight and undernutrition coexisting at the household level have been reported [11, 25, 26, 35].

The DBM at the household level is a complex public health problem [36]. The most important contributing factors of DBM include the place of residence [24, 26], older age of the child (age ≥ 24 months) [25, 26, 37], being a female child [37], maternal older age (age over 30 years) [15, 37], household socioeconomic status [25, 38, 39], richest wealth quintile [15], average birth weight [25], maternal education [15, 37, 38], large family size/household size [11, 37], and more siblings in the household [38].

Maternal height is a useful indicator for predicting children’s risk of developing malnutrition [40–44]. However, its influences on DBM have not been well investigated. Only a few studies have shown that DBM is strongly tied to maternal height [15, 24, 37, 45, 46]. As mentioned, a few pocket studies from Mexico [47], Indonesia [37], Guatemala [45], and Brazil [48] have examined the associations and suggest that short maternal height is associated with a higher risk of DBM. Apart from these examples, studies on the association between maternal stature and DBM in developing countries are rare.

To our knowledge, no studies have documented such an association in Ethiopia. Also, there needs to be more data that have comprehensively examined household-level DBM using a large, pooled dataset in Ethiopia. Previous studies on DBM conducted in Ethiopia have focused on describing the individual-level DBM [35, 49–51], localized in some areas [11, 35], survey specific [25, 26], and focus on the coexistence of maternal overweight/obesity and child stunting or anemia [52]. Considering the above, the aims of the present study were to: (1) determine the prevalence of DBM and (2) examine the association between maternal stature and DBM among mother–child pairs in Ethiopia. Given the national and global targets of achieving food security and improving maternal and child nutrition, this study is paramount in providing factual insights regarding the current status of DBM and designing appropriate preventive strategies in Ethiopia. Additionally, with these pooled data, we better understand maternal stature's influence on the double burden of malnutrition.

## Methods

### Data sources and sampling design

This study utilized data from the four consecutive Ethiopia Demographic and Health Survey (EDHS) (2000–2016), a nationally representative cross-sectional household survey [53–56]. Pooled data on mother–child pairs from the EDHS were included in the study, to explore the prevalence of double burden of malnutrition (DBM). This pooled data analysis also increased the study power, which allowed a full exploration of the effect of maternal height on DBM. In the EDHS, ever-married women aged 15–49 years were interviewed for data on women and children (0–59 months). The survey was designed to be representative at both national and regional levels. The EDHS sampling and household listing methods have been described elsewhere [56]. We used anthropometric indices such as height-for-age, weight-for-height, and weight-for-age to evaluate children’s nutritional status below 5 years of age (0–59 months). In addition, the study used the women’s body mass index (BMI) according to WHO cutoff values [57]. Maternal body mass index (BMI) was classified as underweight (< 18.5 kg/ m^2^), normal (18.5 to < 24.99 kg/m^2^), or overweight/obesity ≥ 25.0 kg/m^2^).

The EDHS collected data on the nutritional status of children by measuring the weight and height of children under the age of 5 years in all sampled households, regardless of whether their mothers were interviewed in the survey or not. Weight was measured with an electronic mother–infant scale (SECA 878 flat) designed for mobile use [56]. Height was measured with a measuring board (ShorrBoard®). Children younger than 24 months were measured lying down on the board (recumbent length), while standing height was measured for all older children.

The three child anthropometric indices used in this study were calculated using growth standards published by the World Health Organization (WHO) in 2006 [58]. The height-for-age index is an indicator of linear growth retardation and cumulative growth deficits in children. Children with height-for-age *Z*-score below minus two standard deviations (− 2 SD) from the median of the WHO reference population are stunted or chronically malnourished. The weight-for-height index measures body mass in relation to body height or length and describes current nutritional status. Children whose *Z*-score is below minus two standard deviations (− 2 SD) from the median of the reference population are considered thin (wasted), or acutely undernourished. Weight-for-age is a composite index of height-for-age and weight-for-height that accounts for both acute and chronic undernutrition. Children whose weight-for-age *Z*-score is below minus two standard deviations (− 2 SD) from the median of the reference population are classified as underweight [58].

### Outcome variable

The primary outcome of this study was DBM, derived from three child anthropometric indices (stunting, wasting, and underweight) and the body mass index (BMI) of their respective mothers. Height-for-age (HAZ), weight-for-height (WHZ), and weight-for-age (WAZ) *z*-scores below − 2 SD of the WHO Child Growth Standard were used to define stunting, wasting, and underweight, respectively [58]. A child who was either stunted, wasted, or underweight and the mother is over-nourished (overweight/ obese) in the same household was considered as having DBM, as used in past studies [15, 25, 59]. Following previous studies, the binary response variable DBM was measured using “normal” and “DBM” response categories. Additionally, the prevalence of overweight/obese mothers and stunted children, overweight/obese mother and wasted children, and overweight/obese mother and underweight child was estimated.

### Main exposure

The main exposure of our study was maternal height. We adopted height cutoffs used by previous studies [37, 60, 61], but subdivided them into three categories. Accordingly, we categorized maternal height as: very short (< 145.0 cm), short (145.0 to 154.9 cm) and normal/tall (≥ 155.0 cm).

### Control variables

Covariates were considered based on the availability of data and previous literature [15, 25, 47, 62–65]. In this study, we included two levels of confounding variables: individual (i.e., child, maternal, and household factors) and community levels. The individual-level covariates included: child factors (child’s age in months, gender, birth order, birth interval, size of child at birth, diarrhea, fever, and ARI), maternal factors (mother’s age, mother’s education, mother's occupation, ANC visit, anemia status, listening to the radio, and watching television), and household-level covariates (wealth index, household size, type of cooking fuel, toilet facility, source of drinking water, household flooring, and time to get a water source). Lastly, the community-level factors include the place of residence (urban or rural) and contextual region of residence (agrarian, pastoralist, and city administration).

### Data analysis

All analyses were carried out using STATA/MP version 14.1 (StataCorp, College Station, TX, USA). The survey command (*svy*) in STATA was used to take into account the sampling design of the survey. Sampling weighting was applied to all descriptive statistics to compensate for the disproportionate allocation of the sample. The weighting technique is explained in full in the EDHS report [56]. Descriptive statistics such as frequencies and percentages were used to present the distribution of all variables.

Given the hierarchical nature of the EDHS data, a multilevel binary logistic regression model was fitted to estimate the association between DBM and maternal height. In this model-building process, we first performed an unadjusted bivariable multilevel analysis between DBM and exposure or each of the covariates. Variables in bivariable analysis with a *p* value < 0.2 were entered in the multilevel multivariable binary logistic regression models. All independent variables associated with the DBM were tested for multicollinearity and there was no evidence of multicollinearity. Following the recommendations of a previous study, five hierarchical models were run [66–68]. Accordingly, five models were fitted: the empty model without any explanatory variable was run to detect the presence of a possible contextual effect (*Model I*), *Model II* (*Model I* + child characteristics), *Model III* (Model II + mothers characteristics), *Model IV* (*Model III* + household characteristics), *Model V* (*Model IV* + community-level characteristics) were fitted. In our analysis, all models assumed a random intercept. Model comparisons were done using the deviance information criteria (DIC) and the model with the lowest DIC value was chosen as the best-fitted model for the data. The intraclass correlation coefficient (ICC) was computed for each model to show the amount of variations explained at each level of modeling. The adjusted odds ratio (AOR) with a 95% confidence interval (CI) and *p* value < 0.05 in the *Model V* multivariable model were used to declare significant determinants of DBM and its association with maternal height. Finally, after controlling all covariates and exposure variables the mean value estimates were presented (the estimates are obtained after the post-estimation command) using the figure representing the predictive probability for DBM and maternal height.

### Ethical consideration

The data used in this study were obtained from the MEASURE DHS Program, and permission for data access was obtained from the measure DHS program through an online request from http://www.dhsprogram.com. The data used for this study were publicly available with no personal identifier. There was no need for ethical clearance as the researcher did not interact with respondents.

## Result

### Characteristics of study participants

Table 1 presents the frequency and the weighted distribution of DBM, overweight/obesity mother–stunted child, overweight/obesity mother–wasted child, overweight/obesity mother–underweight child, and covariates in the study population. In this study, we analyzed data from a total of 33,454 mother–child pairs among whom there were 20,417 (61.0%) normal/tall (≥ 155.0 cm) mothers, 12,265 (36.7%) short (145–155 cm) mothers, and 771 (2.3%) mothers were of very short (< 145.0 cm) stature. The mean maternal height was (156.69 cm ± 6.34). Almost 4 in 10 children belong to the age-group of 36–59 months. Most of the children resided in rural areas (89.2%).

**Table 1:** Socio‐demographic characteristics of the sample population and prevalence of mother–child pairs of double burden of malnutrition by child, maternal, household, and community-level characteristics, EDHS (2000–2016)

Variables	Total (*n*)	Percent (%)	Overweight/obese mother–stunted child, 95% CI	Overweight/obese mother–wasted child, 95% CI	Overweight/obese mother–underweight child, 95% CI	DBM, 95% CI
*Maternal stature*						
Normal/tall (≥ 155 cm)	20,417	61.0	1.37 (1.21–1.54)	0.41 (0.32–0.50)	0.71 (0.61–0.84)	1.70 (1.53–1.89)
Short (145 to 154.9 cm)	12,265	36.7	1.51 (1.29–1.77)	0.20 (0.13–0.31)	0.53 (0.41–0.69)	1.72 (1.48–1.98)
Very short (< 145 cm)	771	2.3	5.52 (3.97–7.64)	0.32 (0.08–1.29)	0.30 (0.19–0.47)	5.70 (4.11–7.84)
*Individual-level characteristics*						
Child factors						
Sex						
Male	17,022	50.9	1.67 (1.48–1.89)	0.39 (0.31–0.51)	0.78 (0.65–0.94)	1.96 (1.75–2.19)
Female	16,431	49.1	1.32 (1.15–1.52)	0.27 (0.20–0.37)	0.62 (0.50–0.75)	1.61 (1.41–1.82)
Age (months)						
< 6	3303	9.9	0.53 (0.32–0.86)	0.67 (0.43–1.03)	0.29 (0.15–0.56)	1.14 (0.82–1.59)
6–11	3519	10.5	0.59 (0.38–0.93)	0.56 (0.35–0.89)	0.56 (0.35–0.89)	1.09 (0.78–1.52)
12–23	6519	19.6	0.96 (0.74–1.24)	0.24 (0.15–0.41)	0.36 (0.23–0.55)	1.16 (0.92–1.47)
24–35	6387	19.1	2.07 (1.74–2.46)	0.32 (0.20–0.49)	0.96 (0.74–1.24)	2.31 (1.95–2.72)
36–59	13,620	40.8	1.95 (1.72–2.21)	0.25 (0.18–0.36)	0.88 (0.73–1.06)	2.16 (1.92–2.43)
Birth order						
First born	5962	17.8	1.09 (0.86–1.40)	0.24 (0.14–0.40)	0.56 (0.40–0.79)	1.37 (1.10–1.71)
2–4	14,420	43.1	1.41 (1.22–1.62)	0.28 (0.21–0.39)	0.55 (0.43–0.69)	1.63 (1.43–1.86)
5 or higher	13,071	39.1	1.82 (1.58–2.08)	0.44 (0.34–0.58)	0.95 (0.79–1.15)	2.17 (1.92–2.46)
Birth interval						
< 33 months	23,093	69.0	1.27 (1.12–1.43)	0.26 (0.21–0.34)	0.58 (0.48–0.69)	1.47 (1.32–1.65)
≥ 33 months	10,360	31.0	2.04 (1.77–2.35)	0.49 (0.37–0.66)	0.98 (0.80–1.20)	2.50 (2.20–2.84)
Size of child at birth						
Larger	10,348	31.0	1.58 (1.35–1.86)	0.23 (0.15–0.35)	0.55 (0.42–0.73)	1.79 (1.54–2.08)
Average	13,113	39.3	1.45 (1.25–1.68)	0.37 (0.27–0.49)	0.68 (0.54–0.84)	1.78 (1.55–2.03)
Small	9897	29.7	1.49 (1.26–1.75)	0.41 (0.29–0.56)	0.88 (0.71–1.09)	1.81 (1.55–2.09)
Currently breast-feeding						
Yes	24,827	74.2	1.04 (0.92–1.19)	0.29 (0.23–0.37)	0.53 (0.44–0.64)	1.29 (1.15–1.45)
No	8626	25.8	2.57 (2.26–2.91)	0.44 (0.32–0.60)	1.09 (0.90–1.33)	2.94 (2.6–3.30)
Full vaccination (*n* = 28,647)						
Yes	5572	19.4	2.15 (1.81–2.55)	0.29 (0.19–0.47)	0.71 (0.53–0.95)	2.43 (2.07–2.85)
No	23,075	80.6	1.15 (1.02–1.31)	0.32 (0.25–0.41)	0.60 (0.50–0.71)	1.41 (1.26–1.59)
Diarrhea						
Yes	5722	17.1	1.21 (0.95–1.55)	0.25 (0.14–0.43)	0.71 (0.51–0.97)	1.43 (1.13–1.79)
No	27,685	82.9	1.56 (1.42–1.72)	0.35 (0.29–0.44)	0.70 (0.61–0.81)	1.86 (1.70–2.04)
Fever						
Yes	6815	20.4	1.21 (0.97–1.51)	0.34 (0.22–0.51)	0.74 (0.64–0.85)	1.50 (1.23–1.83)
No	26,590	79.6	1.57 (1.42–1.74)	0.34 (0.27–0.42)	0.55 (0.39–0.76)	1.85 (1.69–2.04)
ARI						
Yes	1344	4.0	1.78 (1.15–2.74)	0.44 (0.18–1.06)	0.62 (0.29–1.28)	2.23 (1.51–3.28)
No	32,109	96.0	1.49 (1.35–1.63)	0.33 (0.27–0.40)	0.70 (0.62–0.81)	1.77 (1.62–1.93)
Parental factors						
Mother’s age						
< 18	270	0.8	–	0.41 (0.57–2.87)	0.40 (0.05–2.84)	0.41 (0.05–2.88)
18–24	7774	23.2	0.81 (0.62–1.04)	0.18 (0.10–0.31)	0.41 (0.29–0.59)	0.97 (0.77–1.23)
25–34	16,972	50.7	1.57 (1.38–1.77)	0.32 (0.24–0.42)	0.67 (0.56–0.82)	1.81 (1.61–2.03)
≥ 35	8437	25.2	2.06 (1.76–2.41)	0.52 (0.38–0.72)	1.04 (0.83–1.29)	2.54 (2.21–2.93)
Mother’s education						
No education	24,378	72.9	1.27 (1.13–1.43)	0.27 (0.21–0.35)	0.67 (0.57–0.79)	1.50 (1.35–1.67)
Primary	7358	22.0	1.89 (1.58–2.25)	0.38 (0.26–0.57)	0.72 (0.54–0.96)	2.18 (1.84–2.56)
Secondary	1325	3.9	2.29 (1.69–3.12)	0.57 (0.31–1.06)	0.74 (0.43–1.28)	2.82 (2.14–3.72)
Higher	392	1.2	3.67 (2.38–5.63)	1.47 (0.74–2.92)	1.28 (0.61–2.67)	5.16 (3.59–7.38)
Mother’s occupation						
Not working	16,232	48.7	1.58 (1.40–1.79)	0.35 (0.27–0.45)	0.80 (0.67–0.95)	1.89 (1.69–2.12)
Non-agriculture	7011	21.0	2.35 (2.01–2.75)	0.59 (0.42–0.81)	1.02 (0.80–1.29)	2.82 (2.44–3.26)
Agriculture	10,108	30.3	0.66 (0.51–0.86)	0.09 (0.05–0.19)	0.24 (0.15–0.37)	0.75 (0.58–0.96)
Antenatal care (ANC) visit						
None	13,080	57.1	1.04 (0.08–1.25)	0.21 (0.14–0.31)	0.50 (0.38–0.65)	1.22 (1.03–1.43)
1–3	5285	23.1	1.57 (1.25–1.97)	0.42 (0.27–0.65)	0.79 (0.57–1.08)	1.96 (1.60–2.40)
4–7	4054	17.7	2.27 (1.87–2.77)	0.69 (0.48–0.99)	0.98 (0.73–1.33)	2.84 (2.38–3.38)
8+	494	22.2	3.53 (2.45–5.07)	0.75 (0.34–1.68)	0.88 (0.42–1.84)	4.30 (3.09–5.96)
Any anemia (*n* = 23,593)						
Yes	6275	26.6	1.64 (1.35–1.98)	0.37 (0.28–0.48)	0.93 (0.72–1.20)	2.07 (1.76–2.44)
No	17,318	73.4	1.64 (1.45–1.86)	0.50 (0.28–0.48)	0.74 (0.61–0.88)	1.94 (1.73–2.17)
Listening to radio						
Yes	11,515	34.4	1.97 (1.72–2.26)	0.44 (0.33–0.59)	0.81 (0.65–1.04)	2.37 (2.09–2.69)
Not at all	21,929	65.6	1.27 (1.12–1.43)	0.28 (0.22–0.36)	0.65 (0.55–0.77)	1.50 (1.34–1.67)
Watching television						
Yes	5865	17.5	2.83 (2.44–3.28)	0.66 (0.48–0.90)	1.04 (0.81–1.33)	3.42 (2.98–3.91)
Not at all	27,568	82.5	1.18 (1.05–1.33)	0.26 (0.20–0.33)	0.62 (0.53–0.73)	1.40 (1.26–1.55)
Household factors						
Wealth index						
Poor	10,711	45.2	1.15 (0.97–1.37)	0.34 (0.25–0.47)	0.67 (0.54–0.84)	1.46 (1.25–1.70)
Middle	4958	20.9	0.76 (0.52–1.11)	0.17 (0.07–0.37)	0.39 (0.23–0.66)	0.91 (0.64–1.28)
Rich	7999	33.8	2.80 (2.45–3.19)	0.62 (0.47–0.83)	1.14 (0.93–1.41)	3.30 (2.92–3.73)
Household size						
1–4	8042	24.0	1.25 (1.03–1.53)	0.23 (0.15–0.37)	0.58 (0.43–0.77)	1.48 (1.23–1.78)
≥ 5	25,411	76.0	1.58 (1.43–1.75)	0.37 (0.30–0.46)	0.74 (0.64–0.86)	1.89 (1.72–2.07)
Type of cooking fuel (*n* = 32,865)						
Clean fuels	32,507	98.9	5.26 (3.68–7.47)	1.09 (0.49–2.41)	1.61 (0.83–3.06)	1.70 (1.56–1.85)
Solid fuels	357	1.1	1.42 (1.29–1.57)	0.32 (0.26–0.39)	0.69 (0.60–0.79)	6.01 (4.30–8.33)
Toilet facility (*n* = 32, 865)						
Improved	3752	11.4	3.28 (2.82–3.81)	0.80 (0.59–1.09)	1.44 (1.14–1.81)	3.91 (3.41–4.48)
Unimproved	10,443	31.8	1.66 (1.39–1.98)	0.34 (0.23–0.51)	0.73 (0.56–0.95)	1.96 (1.67–2.31)
Open defecation	18,669	56.8	0.93 (0.79–1.08)	0.21 (0.15–0.28)	0.49 (0.40–0.61)	1.11 (0.96–1.27)
Source of drinking water (32,858)						
Improved	12,667	38.5	2.26 (2.01–2.54)	0.52 (0.40–0.66)	1.05 (0.88–1.25)	2.71 (2.43–3.01)
Unimproved	20,190	61.5	0.99 (0.86–1.15)	0.22 (0.16–0.30)	0.48 (0.39–0.60)	1.17 (1.03–1.34)
Household flooring						
Improved	2685	8.0	4.26 (3.68–4.94)	1.05 (0.73–1.36)	1.64 (1.29–2.88)	5.05 (4.41–5.78)
Unimproved	30,761	92.0	1.08 (0.97–1.22)	0.24 (0.18–0.30)	0.56 (0.47–0.66)	1.29 (1.17–1.44)
Time to get a water source						
On–premise	1744	5.2	4.06 (3.35–4.90)	1.14 (0.79–1.64)	1.71 (1.27–2.29)	5.07 (4.28–6.0)
≤ 30 min	19,373	58.3	1.26 (1.10–1.45)	0.19 (0.14–0.28)	0.58 (0.47–0.71)	1.44 (1.27–1.64)
31–60 min	6840	20.6	1.14 (0.90–1.45)	0.32 (0.21–0.51)	0.55 (0.39–0.78)	1.37 (1.10–1.71)
> 60 min	5291	15.9	1.22 (0.97–1.54)	0.33 (0.21–0.52)	0.68 (0.50–0.92)	1.49 (1.22–1.84)
*Community*-*level characteristics*						
Residence						
Urban	3612	10.8	3.99 (3.48–4.57)	1.03 (0.78–1.35)	1.66 (1.34–2.05)	4.74 (4.18–5.36)
Rural	29,841	89.2	1.01 (0.89–1.14)	0.20 (0.15–0.26)	0.51 (0.43–0.61)	1.21 (1.08–1.35)
Region						
Agrarian	18,220	54.5	1.31 (1.15–1.48)	0.33 (0.25–0.42)	0.69 (0.58–0.82)	1.56 (1.39–1.75)
Pastoralist	14,450	43.2	1.11 (0.91–1.37)	0.19 (0.11–0.31)	0.56 (0.41–0.75)	1.30 (1.07–1.58)
City administration	783	2.3	2.96 (2.50–3.50)	0.62 (0.43–0.90)	0.99 (0.74–1.32)	3.52 (3.02–4.10)
Survey year						
EDHS-2000	9785	29.2	1.11 (0.91–1.35)	0.15 (0.08–0.26)	0.46 (0.34–0.63)	1.23 (1.02–1.49)
EDHS-2005	4282	12.8	1.46 (1.12–1.89)	0.26 (0.14–0.48)	0.67 (0.46–0.99)	1.75 (1.38–2.22)
EDHS-2011	9989	29.8	1.44 (1.22–1.70)	0.28 (0.19–0.41)	0.62 (0.48–0.81)	1.65 (1.41–1.92)
EDHS-2016	9340	28.1	1.96 (1.69–2.27)	0.61 (0.46–0.79)	1.02 (0.83–1.25)	2.49 (2.18–2.84)

All values were weighted

### Prevalence of malnutrition

The prevalence of malnutrition is reported in Table 2. The prevalence of stunting, wasting, and being underweight among under-five children in Ethiopia is 47.31% (95% CI 46.77–47.84), 10.95% (95% CI 10.62–11.29), and 31.51% (95% CI 31.01–32.01), respectively. The prevalence of overweight/obese mothers was 3.21% (95% CI 3.03–3.40).

**Table 2:** Prevalence of malnutrition and double burden of malnutrition (DBM) at household level in Ethiopia, EDHS (2000–2016)

Levels of malnutrition	Frequency	Prevalence (%)	95% CI
Stunted child (*n* = 33,564)	15,878	47.31	46.77–47.84
Wasted child (*n* = 33,583)	3679	10.95	10.62–11.29
Underweight child (*n* = 33,729)	10,627	31.51	31.01–32.01
Overweight/obesity mother (*n* = 34,441)	1105	3.21	3.03–3.40
*Double burden of malnutrition at household level*			
Overweight/obesity mother and stunted child (*n* = 33,547)	439	1.31	1.19–1.44
Overweight/obesity mother and wasted child (*n* = 33,566)	77	0.23	0.18–0.28
Overweight/obesity mother and underweight child (*n* = 33,711)	194	0.58	0.51–0.66
Overweight/obesity mother and stunted or wasted or underweight child (*n* = 33,454)	508	1.52	1.39–1.65

All values were weighted

### Prevalence of double burden of malnutrition

Table 2 also presents the weighted prevalence of different forms of the double burden of malnutrition. The prevalence of overweight/obesity mother and stunted children was 1.31% (95% CI 1.19–1.44), while the prevalence of overweight/obesity mother and the wasted child was 0.23% (95% CI 0.18–0.28) and that of overweight/obese mothers and the underweight children was 0.58% (95% CI 0.51–0.66). Overall, the prevalence of DBM was found to be 1.52% (95% CI 1.39–1.65). The prevalence of DBM was significantly higher (5.7%) among the children of women with very short maternal height (< 145 cm). The highest prevalence of the DBM (2.31%, 95% CI 1.95–2.72) occurred among children aged 24–35 months. DBM was highest among women over 35 years (2.54%, 95% CI 2.21–2.93) of age than women in any other age-group. The prevalence of DBM was higher among urban residents (4.74% vs 1.21%).

### Association of DBM and maternal stature

The unadjusted association between DBM and exposure and other study covariates are given in Table 3. The association between DBM and maternal very short height (< 145 cm) was highly significant (*p* value < 0.001) in the unadjusted model. Tables 4 and 5 present the adjusted odds ratio (AOR) with a 95% CI of DBM and maternal height. Our results showed that DBM was positively associated with maternal height adjusted for individual (i.e., child, maternal, household) and community-level covariates. The adjusted multilevel models estimated that compared to the children of tall mothers (height ≥ 155 cm), the odds of DBM was 1.37 times higher among children whose mothers’ height ranged from 145 to 155 cm (AOR: 1.37, 95% CI 1.04–1.80). The odds of DBM was 2.98 times higher among children whose mothers had short stature (height < 145 cm) (AOR: 2.98, 95% CI 1.52–5.86) compared to children whose mothers had tall stature (height ≥ 155 cm).

**Table 3:** Unadjusted association between double burden of malnutrition (DBM) and maternal heights and other study covariates among mother–child pairs in Ethiopia, EDHS (2000–2016)

Variables	DBM Weighted prevalence (95% CI)	Unadjusted OR, 95% CI	*p* value
*Maternal stature*			
Normal/tall (≥ 155 cm)	1.70 (1.53–1.89)	Ref	
Short (145 to 154.9 cm)	1.72 (1.48–1.98)	1.05 (0.87–1.26)	0.610
Very short (< 145 cm)	5.70 (4.11–7.84)	3.76 (2.59–5.44)	< 0.001
*Individual-level characteristics*			
Child factors			
Sex			
Male	1.96 (1.75–2.19)	Ref	
Female	1.61 (1.41–1.82)	0.81 (0.68–0.96)	0.018
Age (months)			
< 6	1.14 (0.82–1.59)	0.52 (0.36–0.74)	< 0.001
6–11	1.09 (0.78–1.52)	0.49 (0.35–0.71)	< 0.001
12–23	1.16 (0.92–1.47)	0.53 (0.41–0.69)	< 0.001
24–35	2.31 (1.95–2.72)	1.07 (0.87–1.32)	0.517
36–59	2.16 (1.92–2.43)	Ref	
Birth order			
First born	1.37 (1.10–1.71)	0.61 (0.47–0.78)	< 0.001
2–4	1.63 (1.43–1.86)	0.74 (0.61–0.89)	0.001
5 or higher	2.17 (1.92–2.46)	Ref	
Birth interval			
< 33 months	1.47 (1.32–1.65)	Ref	
≥ 33 months	2.50 (2.20–2.84)	1.74 (1.46–2.07)	< 0.001
Size of child at birth			
Larger	1.79 (1.54–2.08)	Ref	
Average	1.78 (1.55–2.03)	1.01 (0.81–1.23)	0.979
Small	1.81 (1.55–2.09)	1.03 (0.82–1.28)	0.796
Currently breast-feeding			
Yes	1.29 (1.15–1.45)	Ref	
No	2.94 (2.6–3.30)	2.26 (1.90–2.69)	< 0.001
Full vaccination			
Yes	2.43 (2.07–2.85)	Ref	
No	1.41 (1.26–1.59)	0.58 (0.47–0.71)	< 0.001
Diarrhea			
Yes	1.43 (1.13–1.79)	0.77 (0.60–0.99)	0.045
No	1.86 (1.70–2.04)	Ref	
Fever			
Yes	1.50 (1.23–1.83)	0.82 (0.65–1.02)	0.076
No	1.85 (1.69–2.04)	Ref	
ARI			
Yes	2.23 (1.51–3.28)	1.28 (0.85–1.94)	0.234
No	1.77 (1.62–1.92)	Ref	
Maternal factors			
Mother’s age			
< 18	0.41 (0.05–2.88)	0.16 (0.02–1.12)	0.065
18–24	0.97 (0.77–1.23)	0.37 (0.28–0.49)	< 0.001
25–34	1.81 (1.61–2.03)	0.69 (0.58–0.84)	< 0.001
≥ 35	2.54 (2.21–2.93)	Ref	
Mother’s education			
No education	1.51 (1.35–1.67)	0.61 (0.51–0.73)	< 0.001
Primary and above	2.49 (2.18–2.82)	Ref	
Mother’s occupation			
Not working	1.89 (1.69–2.12)	Ref	
Non-agriculture	2.82 (2.44–3.26)	1.49 (1.23–1.80)	< 0.001
Agriculture	0.75 (0.58–0.96)	0.39 (0.30–0.53)	< 0.001
Antenatal care (ANC) visit			
None	1.22 (1.03–1.43)	Ref	
1–3	1.96 (1.60–2.40)	1.62 (1.24–2.11)	< 0.001
4–7	2.84 (2.38–3.38)	2.36 (1.84–3.02)	< 0.001
8+	4.30 (3.09–5.96)	3.59 (2.43–5.29)	< 0.001
Listening to radio			
Yes	2.37 (2.09–2.69)	Ref	
Not at all	1.50 (1.34–1.67)	0.63 (0.53–0.75)	< 0.001
Watching television			
Yes	3.42 (2.98–3.91)	Ref	
Not at all	1.40 (1.26–1.55)	0.41 (0.34–0.49)	< 0.001
Household factors			
Wealth index			
Poor	1.46 (1.25–1.70)	Ref	
Middle	0.91 (0.64–1.28)	0.61 (0.42–0.90)	0.014
Rich	3.30 (2.92–3.73)	2.19 (1.77–2.70)	< 0.001
Household size			
1–4	1.48 (1.23–1.78)	0.77 (0.62–0.94)	0.014
≥ 5	1.89 (1.72–2.07)	Ref	
Type of cooking fuel			
Clean fuels	1.70 (1.56–1.85)	Ref	
Solid fuels	6.01 (4.30–8.33)	0.27 (0.18–0.39)	< 0.001
Toilet facility			
Improved	3.91 (3.41–4.48)	Ref	
Unimproved	1.96 (1.67–2.31)	0.52 (0.41–0.65)	< 0.001
Open defecation	1.11 (0.96–1.27)	0.29 (0.23–0.35)	< 0.001
Source of drinking water			
Improved	2.71 (2.43–3.01)	Ref	
Unimproved	1.17 (1.03–1.34)	0.43 (0.36–0.51)	< 0.001
Household flooring			
Improved	5.05 (4.41–5.78)	3.88 (3.22–4.68)	< 0.001
Unimproved	1.29 (1.17–1.44)	Ref	
Time to get a water source			
On-premise	5.07 (4.28–6.0)	Ref	
≤ 30 min	1.44 (1.27–1.64)	0.28 (0.22–0.35)	< 0.001
31–60 min	1.37 (1.10–1.71)	0.27 (0.19–0.36)	< 0.001
> 60 min	1.49 (1.22–1.84)	0.29 (0.22–0.39)	< 0.001
*Community-level characteristics*			
Residence			
Urban	4.74 (4.18–5.36)	4.07 (3.39–4.89)	< 0.001
Rural	1.21 (1.08–1.35)	Ref	
Region			
Agrarian	1.56 (1.39–1.75)	Ref	
Pastoralist	1.30 (1.07–1.58)	0.79 (0.62–1.01)	0.059
City administration	3.52 (3.02–4.10)	2.33 (1.88–2.89)	< 0.001
Survey year			
EDHS-2000	1.23 (1.02–1.49)	0.48 (0.38–0.61)	< 0.001
EDHS-2005	1.75 (1.38–2.22)	0.68 (0.51–0.91)	0.008
EDHS-2011	1.65 (1.41–1.92)	0.66 (0.53–0.82)	< 0.001
EDHS-2016	2.49 (2.18–2.84)	Ref

**Table 4:** Adjusted odds ratio estimates on the association between maternal heights and double burden of malnutrition (DBM) among mother–child pairs in Ethiopia, EDHS (2000–2016)

Variables	Model 0: without independent variables	Model 1: Maternal stature and child characteristics AOR (95% CI)	Model 2: model 1 + maternal characteristics AOR (95% CI)	Model 3: model 2 + household characteristics AOR (95% CI)	Model 4: model 3 + community-level factors AOR (95% CI)
*Individual-level characteristics*					
Maternal stature					
Normal/tall (≥ 155.0 cm)		Ref	Ref	Ref	Ref
Short (145 to 154.9 cm)		1.08 (0.87–1.33)	1.26 (0.99–1.60)	1.35 (1.03–1.78)*	1.37 (1.04–1.80)*
Very short (< 145.0 cm)		4.05 (2.64–6.19)**	4.03 (2.41–6.72)**	2.94 (1.49–5.77)*	2.95 (1.50–5.80)*
Child factors					
Sex					
Male		Ref	Ref	Ref	Ref
Female		0.81 (0.66–0.98)*	0.83 (0.66–1.04)	0.78 (0.61–1.02)	0.77 (0.60–1.01)
Age (months)					
< 6		0.98 (0.65–1.47)	0.86 (0.52–1.42)	0.66 (0.37–1.18)	0.59 (0.32–1.05)
6–11		0.91 (0.61–1.35)	0.82 (0.50–1.33)	0.61 (0.35–1.06)	0.53 (0.30–0.95)*
12–23		0.81 (0.59–1.09)	0.74 (0.50–1.09)	0.63 (0.41–0.99)*	0.55 (0.34–0.88)*
24–35		1.33 (1.05–1.69)*	1.27 (0.93–1.73)	1.08 (0.75–1.54)	0.95 (0.65–1.40)
36–59		Ref	Ref	Ref	Ref
Birth order					
First born		0.81 (0.58–1.13)	0.81 (0.52–1.25)	0.65 (0.37–1.14)	0.64 (0.37–1.11)
2–4		0.91 (0.71–1.18)	0.84 (0.62–1.14)	0.77 (0.53–1.01)	0.75 (0.52–1.08)
5 or higher		Ref	Ref	Ref	Ref
Birth interval					
< 33 months		Ref	Ref	Ref	Ref
≥ 33 months		1.46 (1.13–1.88)*	1.10 (0.66–1.82)	1.17 (0.68–2.00)	1.19 (0.69–2.04)
Currently breast-feeding					
Yes		Ref	Ref	Ref	Ref
No		1.89 (1.52–2.35)**	1.64 (1.22–2.19)*	1.56 (1.12–2.19)*	1.56 (1.12–2.19)*
Full vaccination					
Yes		Ref	Ref	Ref	Ref
No		0.62 (0.50–0.77)**	1.63 (1.22–2.19)*	0.99 (0.72–1.36)	1.03 (0.75–1.42)
Diarrhea					
Yes		0.90 (0.68–1.20)	0.87 (0.64–1.20)	0.78 (0.53–1.16)	0.79 (0.53–1.18)
No		Ref	Ref	Ref	Ref
Fever					
Yes		0.85 (0.65–1.10)	0.98 (0.74–1.31)	1.07 (0.76–1.52)	1.09 (0.76–1.54)
No		Ref	Ref	Ref	Ref
ARI					
Yes		1.45 (0.91–2.33)	1.17 (0.68–2.04)	1.22 (0.69–2.17)	1.29 (0.72–2.29)
No		Ref	Ref	Ref	Ref
Maternal factors					
Mother’s age					
< 18			0.29 (0.03–2.34)	0.69 (0.08–5.60)	0.76 (0.09–6.23)
18–24			0.41 (0.22–0.78)*	0.47 (0.23–0.97)*	0.49 (0.24–1.02)
25–34			0.75 (0.45–1.24)	0.91 (0.53–1.57)	0.94 (0.54–1.61)
≥ 35			Ref	Ref	Ref
Mother’s education					
No education			Ref	Ref	Ref
Primary and above			1.27 (0.96–1.69)	1.01 (0.72–1.40)	0.98 (0.70–1.38)
Mother’s occupation					
Not working			Ref	Ref	Ref
Non-agriculture			1.13 (0.87–1.45)	0.99 (0.74–1.34)	0.97 (0.72–1.31)
Agriculture			0.40 (0.27–0.58)**	0.58 (0.37–0.92)*	0.61 (0.39–0.97)*
Antenatal care (ANC) visit					
None			Ref	Ref	Ref
1–3			1.42 (1.05–1.93)*	1.22 (0.85–1.75)	1.12 (0.78–1.62)
4–7			1.56 (1.13–2.17)*	1.24 (0.85–1.83)	1.10 (0.74–1.63)
8+			1.85 (1.15–3.00)*	1.21 (0.68–2.16)	1.08 (0.60–1.94)
Listening to radio					
Yes			Ref	Ref	Ref
Not at all			0.87 (0.67–1.13)	1.05 (0.78–1.41)	0.97 (0.72–1.33)
Watching television					
Yes			Ref	Ref	Ref
Not at all			0.63 (0.47–0.85)*	1.22 (0.83–1.78)	1.26 (0.84–1.87)
Household factors					
Wealth index					
Poor				Ref	Ref
Middle				0.53 (0.30–0.94)*	0.55 (0.32–0.98)*
Rich				1.15 (0.75–1.74)	1.08 (0.71–1.67)
Household size					
1–4				0.99 (0.70–1.39)	0.95 (0.67–1.34)
≥ 5				Ref	Ref
Type of cooking fuel					
Clean fuels				Ref	Ref
Solid fuels				0.92 (0.53–1.59)	1.06 (0.61–1.86)
Toilet facility					
Improved				Ref	Ref
Unimproved				0.92 (0.64–1.32)	0.95 (0.65–1.37)
Open defecation				0.65 (0.41–1.02)	0.68 (0.43–1.07)
Source of drinking water					
Improved				Ref	Ref
Unimproved				0.75 (0.55–1.03)	0.80 (0.57–1.12)
Household flooring					
Improved				1.87 (1.23–2.84)*	1.60 (1.04–2.47)*
Unimproved				Ref	Ref
Time to get a water source					
On-premise				Ref	Ref
≤ 30 min				0.59 (0.39–0.89)*	0.71 (0.46–1.08)
31–60 min				0.57 (0.35–0.96)*	0.70 (0.42–1.18)
> 60 min				0.72 (0.44–1.19)	0.88 (0.53–1.48)
*Community-level characteristics*					
Residence					
Urban					1.75 (1.11–2.77)*
Rural					Ref
Region					
Pastoralist					Ref
Agrarian					1.11 (0.77–1.59)
City administration					1.18 (0.74–1.87)
Survey year					
EDHS-2000					0.81 (0.63–1.93)
EDHS-2005					0.78 (0.51–1.21)
EDHS-2011					0.67 (0.48–0.93)*
EDHS-2016					Ref
Random effect					
Variance (SE)	0.5259 (0.0052)**	0.5844 (0.0068)**	0.2060 (0.0169)*	0.0629 (0.0797)*	0.04608 (0.1064)*
Log-likelihood ratio (LL)	− 2718.321	− 2105.148	− 1514.540	− 1113.319	− 1107.582
Deviance	5436.642	4210.297	3029.08	2226.63	2215.16
ICC (%)	13.78	15.08	5.89	1.87	1.38
AIC	5440.64	4244.297	3085.08	2304.64	2303.16
BIC	5457.30	4383.264	3305.03	2596.368	2632.29

*EDHS* Ethiopian Demographic and Health Survey, *AIC* Akaike’s information criterion, *BIC* Bayesian information criterion, *ICC* Inter-cluster correlation, *AOR* Adjusted odds ratio

*p < 0.005; **p < 0.001

**Table 5:** Summary of the association between maternal height and double burden of malnutrition (unadjusted and adjusted odds ratios with 95% CI and absolute probabilities with 95% CI)

	DBM, 95% CI	Unadjusted		Adjusted^a^	
		Unadjusted OR, 95% CI	Absolute probabilities (95% CI)	Adjusted OR, 95% CI	Absolute probabilities (95% CI)
*Maternal stature*					
Normal/tall (≥ 155 cm)	1.70 (1.53–1.89)	Ref	0.017 (0.015–0.018)	Ref	0.016 (0.014–0.019)
Short (145 to 154.9 cm)	1.72 (1.48–1.98)	1.05 (0.87–1.26)	0.017 (0.014–0.019)	1.37 (1.04–1.80)*	0.022 (0.018–0.027)
Very short (< 145 cm)	5.70 (4.11–7.84)	3.76 (2.59–5.44)*	0.057 (0.038–0.075)	2.98 (1.52–5.86)*	0.046 (0.019–0.073)

^a^Model adjusted for individual- and community-level variables

*p < 0.05

Table 5 summarizes unadjusted, adjusted odds ratios and absolute probability of DBM. Marginal effects show the change in probability when the predictor or independent variable increases by one unit. The change in probability of DBM when maternal height goes from normal to short increases by 2.2 percentage and is significant. Similarly, the change in probability when maternal height goes from short to very short increases by 4.6 percentage points, which is also significant.

**Fig. 1:**
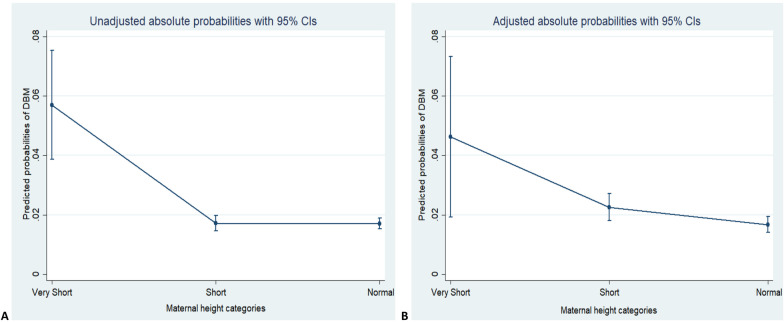
Unadjusted (**A**) and adjusted (**B**) absolute probabilities of DBM and maternal height

Figure 1 shows the predicted probabilities along with their 95% confidence interval. The predicted probability of DBM was on the “*Y axis*” and maternal height was on the “*X axis*.” The fitted line increases from right to left, indicating that as maternal height decreases from normal to very short, the probability of DBM increases.

## Discussion

The concept of the double burden of malnutrition (DBM) at the household level is not well understood in Ethiopia. To our knowledge, this is the first comprehensive assessment undertaken: (a) to determine the prevalence of DBM and (b) to examine the associations between DBM and maternal height in Ethiopia. The prevalence of DBM was below 2%. Our results showed that DBM was strongly associated with maternal height after adjusting for potential individual and community-level covariates.

In this study, the overall prevalence of DBM was 1.52%. However, the DBM increased with age in women after 35 years and increased with urbanization. The current finding in India indicates a rising concern about DBM as the country goes through a perfect wave of changes in dietary patterns and physical activity due to urbanization and economic development [69]. Ethiopia is implementing policies that will allow the country to achieve lower-middle-income status. Henceforth, the coexistence of multiple forms of malnutrition in households will likely increase in the coming years. The double burden of malnutrition has also been linked to a high level of food insecurity and a higher prevalence of infection, combined with rapid population growth and urbanization, which may lead to an increase in the prevalence of DBM [19].

The existence of a double burden of malnutrition in the same household was reported in different low-income settings such as in Bangladesh [16, 70], Indonesia [37], Kenya [22], Nepal [15], and India [59]. Few studies have also reported Ethiopia's household-level double burden of malnutrition [11, 25, 26]. The observed prevalence of DBM was lower than the finding from a study in Nepal, 6.6% [15], and studies from Bangladesh, 6·3%, [70] and, 4.9%, [71]. The low-level prevalence of DBM might be due to the low proportion of women who are overweight or obese in the country. In Ethiopia, however, the proportion of women who are overweight or obese has increased over time from 3% in 2000 to 8% in 2016 [56]. In the same period, however, the prevalence of overweight/obesity increased from 6.5 to 22.1% between 2001 and 2016 among women of reproductive age (15–49 years) [72] in Nepal. In Bangladesh, the prevalence of overweight was about 29% and the rate of obesity was approximately 11% among women of reproductive age [73]. Another study from Bangladesh reported increases in the prevalence of overweight and obesity from 2004 to 2014 as follows: the prevalence of overweight increased from 11.4% in 2004 to 25.2% in 2014, and the prevalence of obesity increased from 3.5% to 11.2% over the same period of time [74]. The observed increase in mothers’ overweight or obesity was stated to be associated with the nutrition transition situation [34].

As documented in the current study, the prevalence of DBM is low in Ethiopia compared to other related low-income settings. Another possible explanation for this phenomenon relates to differences in the urbanization of society, maternal nutrition status, socioeconomic development, and sociocultural factors, which may lead to alterations in food consumption habits and accelerate the occurrence of DBM.

Prior evidence from 126 low-income and middle-income countries (LMICs) revealed that the prevalence of household-level DBM ranged from 3 to 35%, with the most prevalent being maternal overweight/obesity and child stunting [2]. In our analysis, the prevalence of overweight/obese mother–stunted child pairs was 1.31%. The prevalence was closely comparable with a study finding from Nepal 1.54% [14]. However, our finding was much lower than the prevalence rates reported from Bangladesh at 4.10%, Pakistan at 3.93%, and Myanmar at 5.54% [14]. Additionally, a much higher prevalence of overweight/obese mother–stunted child was observed in Bangladesh at 24.5% [16], in Benin at 11.5% [75], and in Kenya at 20% [22]. Similarly, the prevalence of overweight/obese mothers and wasted or underweight children was lower than in related studies from Kenya [22] and Bangladesh [70]. The low prevalence of overweight/obese mothers with stunted children in Ethiopia could be attributed to the high burden of stunted children in rural areas and the high prevalence of maternal overweight/obesity in urban areas. According to the most recent 2019 Ethiopian Mini Demographic and Health Survey, childhood stunting remained stagnant at 37% (74.65% live in rural areas) and 12% of children under age 5 are severely stunted [29]. The majority of overweight or obese women are also found in urban areas, and the prevalence of overweight/obesity has increased significantly from 10.9% in 2000 to 21.4% in 2016 [31]. It is also worth noting that policy differences, as well as other commitments to combating malnutrition, as well as factors such as maternal nutrition status, socioeconomic development, and sociocultural factors, may account for variations in the prevalence of DBM.

It has already been reported that maternal stature is linked with adverse child and maternal health outcomes [44, 60, 61, 76–79]. Several studies have also examined the association of maternal height with child malnutrition [40, 42, 43, 80]. In our analysis, DBM was significantly associated with the mother’s height. The adjusted models estimated that compared to the children of tall mothers (height ≥ 155.0 cm), the odds of DBM significantly increased by about 1.37 times among the children of the mothers with 145.0 to 155.0 cm height. Similarly, the odds of DBM was 2.98 times higher for the children of the very shortest mothers (height < 145.0 cm) compared to the children of tall mothers (height ≥ 155.0 cm). This result is consistent with Sunuwar et al., finding in Nepal [15] which reported that short stature in mothers was strongly associated with the risk of DBM compared to mothers of normal height. These linkages also align with previous studies from Indonesia and Bangladesh [37], Mexico [47], Guatemala [81], and Brazil [48] reported that short maternal stature increases the risk of the double burden of malnutrition. Several factors and pathways may have contributed to and explained this association: (1) Body mass index (BMI) gain was significantly higher in short-statured women [82], (2) women of short stature are more likely to have undernourished children than women of normal stature [34, 76], (3) maternal height influences offspring linear growth over the growing period [42], (4) it has been also noted that women with short stature were more likely to suffer from chronic degenerative diseases and subsequently have stunted children than the women of normal stature [34], and (5) stunting is an intergenerational phenomenon passed down from mother to child and contributes to small for gestational age babies. As a result, being a very short or short mother may have carried one or more of the identified risks amplifying the likelihood of experiencing the DBM. This study highlights the importance of developing programs and policies that address the nutrition needs of short-statured mothers in order to break the vicious intergenerational cycle of malnutrition under the same roof.

The strength of this study lies in the robust analytical and statistical methods used. Our findings contribute significantly to knowledge by being the first to investigate the relationship between maternal stature and DBM in the Ethiopian context. Additionally, because we used a nationally representative dataset, the findings of this study are generalizable to similar low-income settings. Nonetheless, our study has some limitations, and the findings should be interpreted with caution. First, the nutritional status of the mother was assessed using BMI. BMI is less accurate than other methods such as waist–hip ratio and skinfold thickness methods to assess the type of overweight/obesity. Second, data on maternal overweight/obesity such as dietary intake, physical activity level, and health status were unavailable. Third, the study could not establish a causal pathway of the association between explanatory and dependent variables due to the cross-sectional nature of the data. Fourth, because some of the independent variables were self-reported, there may have been some recall and social desirability bias, which is beyond the control of the current study. Finally, considering the skewed distribution of the DBM data findings should be interpreted with caution.

## Conclusion

Our study findings show a low prevalence of double burden of malnutrition among mother–child pairs in Ethiopia. Mothers with short and very short stature were more likely to suffer from the double burden of malnutrition. This link between short maternal height and DBM may imply that high-risk mothers (those who are short or very short in stature) should be prioritized for sufficient nutritious food supply and optimal nutrition to break the vicious cycle of malnutrition that exists under the same roof. Again, existing nutrition interventions must make significant and concerted efforts to combat the growing concern of DBM in Ethiopia.

## Data Availability

The datasets analyzed during the current study are available on the Measure DHS Web site https://dhsprogram.com after formal online registration and submission of the project title and detailed project description.

## Abbreviations

AOR: Adjusted odds ratio
ANC: Antenatal care visits
BMI: Body mass index
CI: Confidence interval
DBM: Double burden of malnutrition
EDHS: Ethiopian Demographic and Health Surveys
WHO: World Health Organization
